# Development and Clinical Evaluation of Spring-Assisted Standing Training for Individuals with Spinal Cord Injury: A Safety and Feasibility Study

**DOI:** 10.3390/jcm14196767

**Published:** 2025-09-25

**Authors:** Yukiyo Shimizu, Hideki Kadone, Kai Sasaki, Masashi Yamazaki, Yasushi Hada, Kenji Suzuki

**Affiliations:** 1 Department of Rehabilitation Medicine, Institute of Medicine, University of Tsukuba, Tsukuba 305-8575, Japan; y-hada@md.tsukuba.ac.jp; 2 Department of Cybernics Medicine, Institute of Medicine, University of Tsukuba, Tsukuba 305-8575, Japan; kadone@md.tsukuba.ac.jp; 3 Qolo Inc., Tsukuba 305-8575, Japan; kai.addaption@gmail.com; 4 Department of Orthopaedic Surgery, Institute of Medicine, University of Tsukuba, Tsukuba 305-8575, Japan; masashiy@tsukuba-seikei.jp; 5 Institute of Systems, Information and Engineering, University of Tsukuba, Tsukuba 305-8575, Japan; kenji@ieee.org

**Keywords:** trunk and lower limb impairment, standing training, spinal cord injury, rehabilitation technology

## Abstract

**Background/Objectives:** Standing training is essential for individuals with spinal cord injury (SCI), yet maintaining regular practice after acute rehabilitation remains challenging. To address the need for more practical and accessible standing equipment, we developed a novel spring-assisted standing training device designed to overcome barriers to regular standing practice. This study aimed to assess the safety and feasibility of our newly developed device in individuals with SCI. **Methods:** Six participants with chronic SCI (neurological level of injury T4-L3, American Spinal Injury Association Impairment Scale A-C; 2 females, mean age 41.7 ± 13.4 years) underwent a single session using our chair-based device incorporating passive gas spring mechanisms. We designed this device to enable independent sit-to-stand transitions without electrical power or complex controls. Primary outcomes included safety (adverse events) and feasibility (number of repetitions, Modified Borg Scale). Changes in Modified Ashworth Scale (MAS) scores were assessed as exploratory measures. **Results:** All participants successfully completed training without adverse events. Repetitions ranged from 5 to 60 (median 37), with Modified Borg Scale ratings of 0–4. Notably, the participant with T4 complete injury performed the training without requiring trunk orthosis, demonstrating the device’s inherent stability. MAS sum scores showed a reduction from median 8.75 to 4.25, though this did not reach statistical significance (*p* = 0.13). **Conclusions:** Our newly developed spring-assisted standing training device proved safe and feasible for individuals with SCI, including those with complete thoracic injuries. The device successfully enabled independent sit-to-stand transitions with low perceived exertion, potentially addressing key barriers to regular standing practice and offering a practical rehabilitation solution.

## 1. Introduction

Spinal cord injury (SCI) represents one of the most devastating neurological conditions affecting human mobility and independence. According to the World Health Organization (2024) [1], approximately 15.4 million people globally were living with SCI in 2021, with the condition accounting for over 4.5 million years lived with disability. In Japan alone, over 6200 new cases occur annually [2]. People with SCI face numerous secondary health complications including spasticity, chronic pain, pressure ulcers, respiratory complications, and osteoporosis. Without appropriate management, these complications often lead to premature mortality. The socioeconomic impact is equally profound, with unemployment rates exceeding 60% among adults with SCI and substantial barriers to educational participation for children [1].

The resulting trunk and lower limb dysfunction fundamentally alter an individual’s ability to perform daily activities, with standing and walking becoming difficult or impossible depending on injury level and severity. Most individuals with SCI require wheelchairs for daily mobility, leading to a lifestyle of prolonged sitting that carries multiple health risks. These include progressive joint contractures, decreased bone mineral density, metabolic dysfunction, and increased cardiovascular disease risk [3,4,5]. Physical consequences are compounded by social barriers including differences in eye level during interactions, limited reach for daily activities, and reduced participation in standing-oriented social activities [6,7,8].

The biomechanical demands of sit-to-stand movement are substantial, requiring coordinated activation of multiple muscle groups and significant power generation from hip and knee extensors [9]. The movement involves four distinct phases: flexion-momentum, momentum transfer, extension, and stabilization [10], with the momentum transfer phase generating peak knee moments exceeding 1.5 times body weight [11]. For individuals with SCI who lack voluntary control of these muscle groups, these mechanical demands cannot be met without external assistance, making independent standing impossible.

Given these profound challenges, providing opportunities for supported standing becomes critically important for individuals with SCI. The Multidisciplinary Association of Spinal Cord Injury Professionals strongly recommends standing exercises 3–5 times per week for 30–60 min per session, recognizing that regular standing practice provides essential physiological benefits including maintenance of bone mineral density, preservation of joint range of motion, reduction in muscle spasticity, improvement in bowel function, and enhancement of cardiovascular responses [3]. These benefits are particularly crucial for individuals with SCI, as prolonged wheelchair use without standing opportunities can accelerate the development of secondary complications that further compromise health and quality of life.

Regular standing may also help prevent or manage conditions such as osteoporosis, pressure ulcers, and cardiovascular deconditioning that significantly impact quality of life and survival in this population [12]. However, translating these recommendations into practice faces numerous obstacles. Conventional standing interventions typically rely on static equipment such as standing frames or tilt tables that maintain fixed positions without facilitating dynamic movement patterns. While these devices enable weight-bearing in an upright position, they fundamentally lack the repetitive sit-to-stand transitions that could provide additional neuromuscular stimulation and functional benefits. Orthotic devices offer another option but require significant upper extremity strength, considerable preparation time, and often necessitate caregiver assistance, making them impractical for regular daily use [13].

Recent technological advances have introduced powered exoskeletons that can provide active assistance for standing and walking. These sophisticated devices use motors, sensors, and computer control to support upright mobility. However, their implementation faces significant barriers including high acquisition and maintenance costs, regulatory restrictions that typically limit use to supervised clinical settings, and availability only in specialized rehabilitation centers with trained personnel [14,15].

In practice, these devices remain facility-based interventions, requiring individuals to travel to specialized centers for each training session, which creates ongoing time and transportation barriers. Furthermore, many powered exoskeletons are designed primarily for overground walking rather than repetitive sit-to-stand training, potentially missing the specific benefits of this fundamental movement pattern that forms the basis of many daily activities.

Japan’s healthcare system presents unique challenges for long-term standing practice. While government-supported medical rehabilitation is limited to 180 days post-onset [16], subsequent care transitions to long-term support services that focus primarily on maintaining daily living activities rather than intensive therapeutic interventions. This shift from medical rehabilitation to life support services creates a gap in accessing specialized standing training, particularly as functional improvements and prevention of secondary conditions require ongoing therapeutic intervention throughout the chronic phase of SCI. The substantial cost of standing equipment, often not fully covered by insurance, places additional financial burden on individuals already facing significant medical expenses. Geographic disparities in specialized rehabilitation facilities mean many individuals must travel long distances for therapy, adding time and transportation costs to existing barriers [17].

Recognizing these systemic challenges from clinical practice in rehabilitation medicine, our multidisciplinary team at the University of Tsukuba has been developing practical solutions to bridge the gap between ideal standing recommendations and real-world constraints. Working with individuals with SCI and collaborating across medical and engineering disciplines, we identified critical disconnects between what clinical guidelines recommend and what patients can implement in their daily lives. This clinical insight led to the development of a spring-assisted standing device using passive gas spring mechanisms to facilitate sit-to-stand transitions [18]. The design philosophy prioritizes simplicity and user autonomy, aiming to reduce costs compared to complex powered systems while leveraging biomechanical principles where forward trunk lean shifts the center of gravity to reduce lower extremity demands and maintain stability.

To understand barriers to standing practice, we surveyed 125 wheelchair users including 47 with SCI [8]. Results revealed that while 76.8% recognized health benefits from standing, only 31.9% of SCI individuals were engaged in standing training, with barriers including difficulty accessing medical institutions (27.8%) and challenges with orthotic devices (19.4%).

These low adherence rates have serious health implications. Sedentary behavior is increasingly recognized as an independent risk factor for cardiovascular disease and premature mortality in the general population [19,20]. For individuals with SCI, these risks are amplified, with cardiovascular disease emerging as a leading cause of death in the chronic phase. The combination of low training adherence and elevated health risks creates an urgent need for practical solutions that can overcome existing barriers.

This study represents the first clinical evaluation of spring-assisted standing training using a device specifically designed for rehabilitation settings. We aimed to assess the immediate safety and feasibility through a single-session evaluation in individuals with chronic SCI, hypothesizing that mechanical assistance from gas springs would enable safe standing training even for those with complete thoracic injuries. As an exploratory objective, we examined acute changes in muscle tone immediately following the intervention. While this preliminary investigation focused on immediate effects from a single training session, it seeks to establish safety parameters before proceeding to longer-term studies and to inform development of accessible standing solutions that can bridge the gap between clinical recommendations and real-world practice.

## 2. Materials and Methods

### 2.1. Participants and Study Design

This was a single-session, pre-post intervention study conducted at the University of Tsukuba Hospital. The study was approved by the Institutional Review Board of the Faculty of Engineering, Information, and Systems at the University of Tsukuba (approval no. 2019R300-2, approved on 3 September 2020) and conducted in accordance with the Declaration of Helsinki. All participants provided written informed consent.

Six individuals with chronic SCI (>6 months post-injury) were recruited from the outpatient rehabilitation clinic. Inclusion criteria were inability to stand or maintain standing independently and regular wheelchair use. Exclusion criteria included severe contractures, active pressure ulcers, recent fractures, uncontrolled autonomic dysreflexia, and cardiovascular instability. The enrolled participants had neurological levels ranging from T4 to L3, with AIS grades A–C.

### 2.2. Device

**Figure 1:**
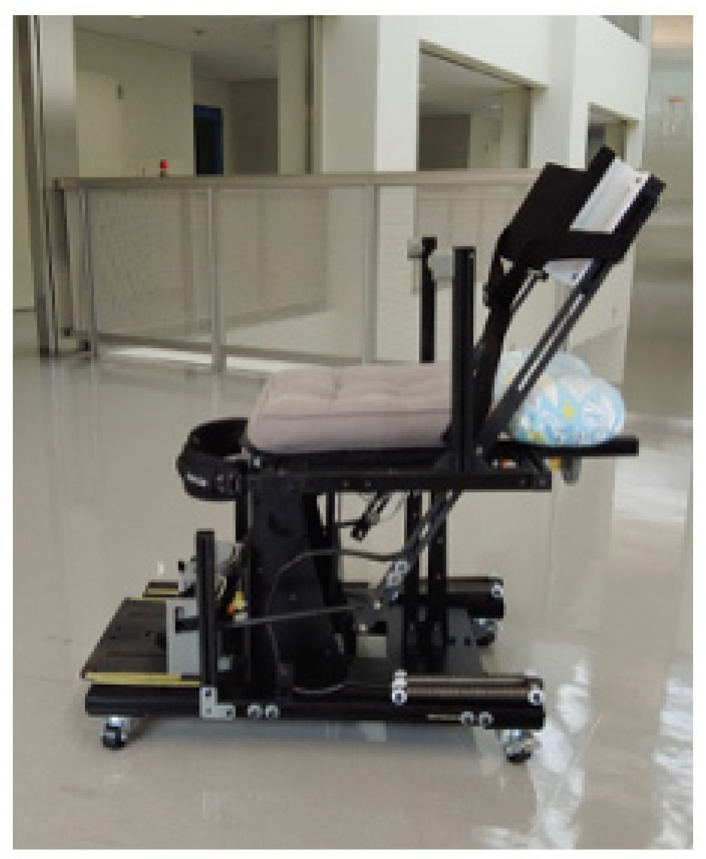
Initial R1A prototype of the spring-assisted standing device with Pelvi.Loc knee supports (RGK Ltd., Burntwood, UK) in sitting position.

**Figure 2:**
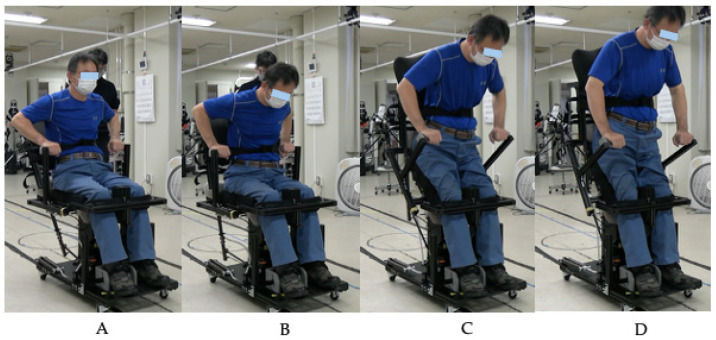
Sequential demonstration of sit-to-stand transition using the R1A prototype. (**A**) Initial sitting position with hands positioned on armrests, feet placed on footplates. (**B**) Initiation phase showing forward trunk lean that engages the gas spring mechanism. (**C**) Mid-transition phase demonstrating progressive knee and hip extension with gas spring assistance while maintaining forward trunk position for stability. (**D**) Final standing position achieved with full knee extension and upright posture.

The spring-assisted standing device was modified from the Qolo technology [18] for clinical training use. The initial R1A prototype (Figure 1) featured passive gas springs that provide progressive assistance during sit-to-stand transitions. The design utilizes a natural forward trunk lean movement pattern: as users lean forward, this motion engages the gas springs, which then provide progressive assistive force throughout the transition, reducing the required knee extensor force [10]. The chair-based design includes adjustable foot plates to accommodate different body sizes. Prior to testing with participants with SCI, we identified knee instability issues with the original Pelvi.Loc knee pads (RGK Ltd., Burntwood, UK) and replaced them with custom-designed knee supports. The complete sit-to-stand sequence using the modified R1A is shown in Figure 2.

### 2.3. Procedures

Following baseline assessment, participants were positioned in the device with appropriate adjustments. After 2–3 practice transitions, they performed repeated sit-to-stand-to-sit cycles at their own pace for up to 20 min. Participants received standardized instructions to lean forward to stand and backward to sit. A physiatrist and one or two engineers monitored continuously but provided no physical assistance unless safety concerns arose.

Primary outcomes included adverse events and feasibility measures (number of repetitions completed and Modified Borg Scale ratings). Perceived exertion was assessed using the Modified Borg Scale (0–10), which has been validated for clinical use [21] and specifically in individuals with SCI [22]. This scale ranges from 0 (none) through 0.5 (very, very light), 1 (very light), 2 (light), 3 (moderate), 4 (a little intense), 5 (intense), 7 (very intense), 9 (very, very intense) to 10 (maximum).

As an exploratory measure, the Modified Ashworth Scale (MAS) [23,24] was assessed bilaterally for six lower limb movements (hip flexion/abduction, knee flexion/extension, ankle dorsiflexion/plantarflexion) before and immediately after the intervention. The MAS grades muscle tone from 0 (no increase in muscle tone) to 4 (affected parts rigid in flexion or extension), with an additional 1+ grade for slight increase in tone with catch followed by minimal resistance. Scores were summed (range 0–60, with 1+ = 1.5). We included this assessment based on our previous report demonstrating the sensitivity of MAS for detecting immediate effects of movement interventions in individuals with SCI [25].

### 2.4. Statistical Analysis

Analyses were performed using JMP Student Edition 18. Descriptive statistics summarized safety and feasibility outcomes. For the exploratory MAS scores and Borg scale ratings, the Wilcoxon signed-rank test was used due to small sample size and non-normal distribution. Significance was set at *p* < 0.05. All participants who received the intervention were included in the analysis.

## 3. Results

### 3.1. Participant Characteristics

Six participants (4 males, 2 females) with chronic SCI completed the study. Mean age was 41.7 ± 13.4 years (range: 23–56). Neurological levels ranged from T4 to L3, with five participants classified as AIS A and one as AIS C. All participants were regular wheelchair users. Detailed participant characteristics are presented in Table 1, with individual joint-specific MAS scores provided in Supplementary Table S1.

### 3.2. Safety Outcomes

All participants completed the training session without adverse events. No episodes of autonomic dysreflexia, orthostatic hypotension, excessive pain, or other safety concerns were reported during or after the intervention. No skin integrity issues were observed at device contact points.

### 3.3. Feasibility Outcomes

The number of sit-to-stand repetitions varied widely among participants, ranging from 5 to 60 with a median of 37 repetitions. Modified Borg Scale ratings remained low throughout the intervention (pre: median 0.5, range 0–4; post: median 3, range 1–4), with no significant change (*p* = 0.19), indicating minimal to light perceived exertion. Notably, the participant with T4 complete injury successfully performed 5 repetitions without requiring trunk orthosis support, demonstrating the device’s inherent stability. All participants achieved independent transitions without physical assistance after the initial practice trials.

### 3.4. Observational Findings

During the training sessions, several device-related observations were noted. The elevated base height of the R1A prototype created transfer difficulties, with participants requiring additional time and effort to position themselves from their wheelchairs. The handle diameter was reported as too large for comfortable gripping by multiple participants. Handle grip patterns varied based on participant characteristics, with taller individuals and those with higher neurological injury levels (T4–T6) tending to grip the handles more proximally compared to those with lower injury levels.

### 3.5. Exploratory Outcome: Changes in Muscle Tone

Exploratory analysis of MAS sum scores showed a reduction from a median of 8.75 (range: 0–12.5) pre-intervention to 4.25 (range: 0–8) post-intervention. While this reduction did not reach statistical significance (Wilcoxon signed-rank test, *p* = 0.13), four of six participants demonstrated reductions in muscle tone. Among the five participants who had spasticity at baseline (*n* = 5), four showed reductions ranging from 4.0 to 10.5 points, while one participant (ID2) maintained the same score. The participant without baseline spasticity (ID3) remained at 0. The most substantial reduction was observed in the participant with the highest baseline MAS score (12.5 to 2). Detailed individual responses are presented in Table 1 and Supplementary Table S1.

### 3.6. Device Development Based on Study Findings

**Figure 3:**
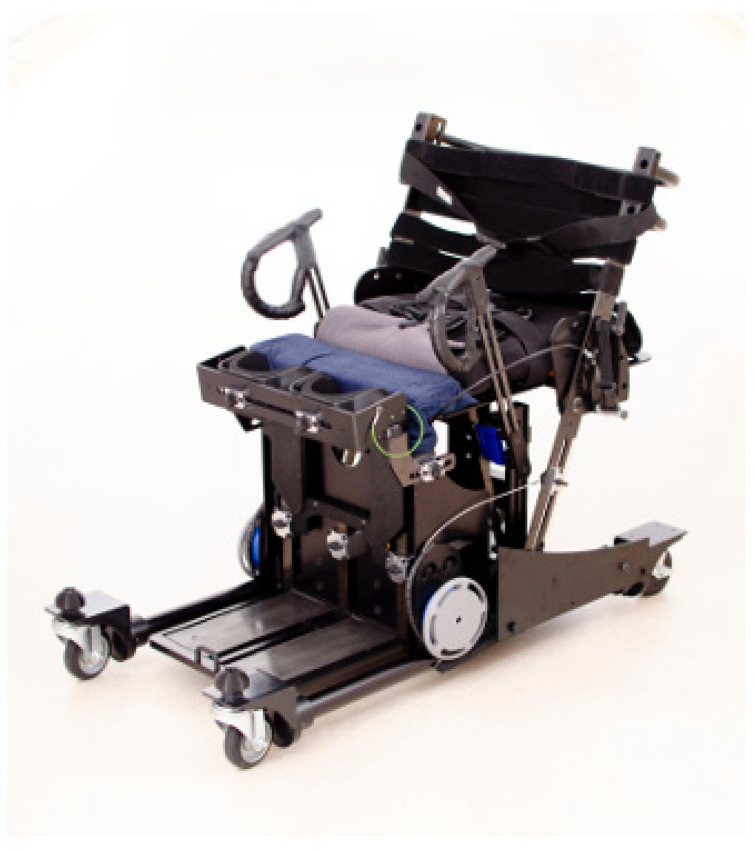
R1B prototype featuring modified handles with reduced diameter and enhanced knee pads with adjustable positioning based on observations from R1A testing.

**Figure 4:**
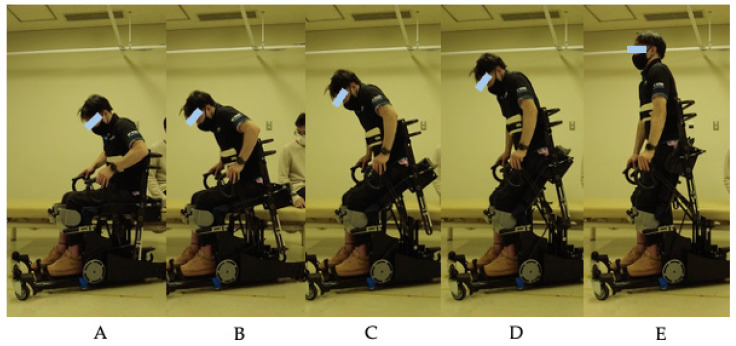
Sequential demonstration of sit-to-stand transition using the R1B prototype with enhanced knee supports and modified handles. (**A**) Starting position showing stable sitting posture. (**B**,**C**) Progressive forward lean phases engaging the gas spring mechanism. (**D**) Mid-to-late transition phase with custom-designed knee supports providing stability. (**E**) Complete standing position achieved independently. The sequence demonstrates successful implementation of the R1B design modifications.

Based on the observational findings from this study, iterative design improvements were implemented. The R1B prototype (Figure 3) incorporated modified handles with reduced diameter and enhanced knee pads with adjustable positioning to address the stability and grip issues identified during R1A testing. Four of the six participants also tested the R1B prototype (Table 2). Figure 4 demonstrates the improved functionality, showing a participant comfortably gripping the modified handles and achieving smooth transitions from sitting to standing with the enhanced knee support system.

**Figure 5:**
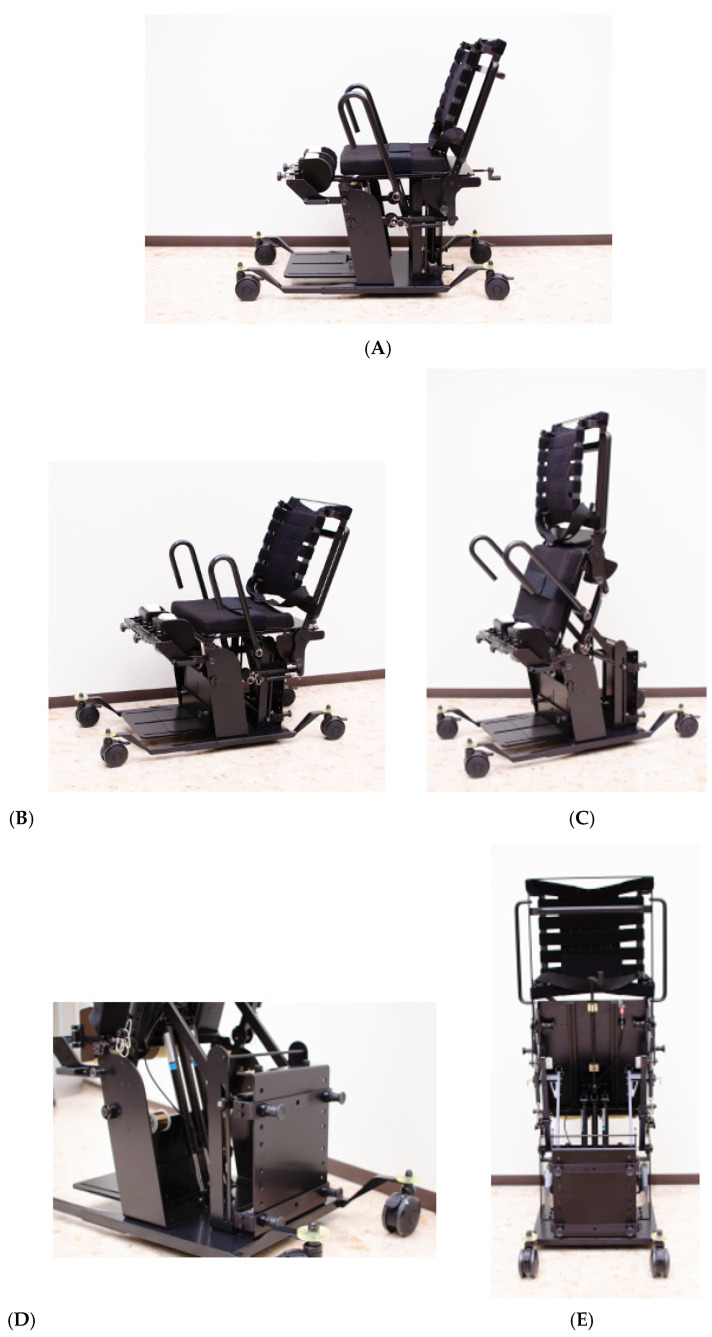
R1C model with comprehensive design improvements based on collective findings from R1A and R1B testing. (**A**) Side view in sitting position showing lowered base design for easier transfers. (**B**,**C**) Sit-to-stand transition sequence: (**B**) sitting position with more upright backrest; (**C**) standing position achieved through forward body lean with spring assistance. (**D**) Oblique rear-left view showing the gas spring assembly and enhanced knee support system. (**E**) Rear view displaying the three gas springs arrangement and rear-mounted handle for assistant level adjustment.

Based on the collective findings from both R1A and R1B testing, the R1C model (Figure 5) features multiple improvements: a lowered base design that facilitates easier wheelchair-to-device transfers, a more upright backrest compared to the reclined position in R1A and R1B models that enables stable sitting without requiring upper extremity support, and handles that allow users to grip at any position along their length to accommodate individual preferences and anthropometric differences. Additionally, R1C includes a rear-mounted handle that allows physicians and therapists to easily adjust the level of assistance provided, enabling adaptation to users with varying physical conditions including different body sizes and degrees of paralysis.

## 4. Discussion

This preliminary study demonstrated that spring-assisted standing training is safe and feasible for individuals with chronic SCI across different injury levels. The absence of adverse events across all participants, including those with complete injuries as high as T4, represents a critical finding for clinical implementation. Individuals with high thoracic SCI face particular risks during standing activities, including autonomic dysreflexia and orthostatic hypotension [26].

The successful participation of the T4 complete injury participant without trunk support is particularly noteworthy. Traditional standing interventions for individuals with high thoracic injuries often require additional precautions and supportive devices [26]. Interestingly, the same systematic review noted that exercise training, including passive leg movements, has the potential to improve orthostatic tolerance by stabilizing cardiovascular responses. The repetitive nature of our standing training protocol may contribute to such cardiovascular adaptations, though this requires further investigation.

The biomechanical advantages of our spring-assisted design merit further discussion. Following principles established in the development of standing mobility devices [18], our device utilizes passive gas springs that provide progressive mechanical assistance during sit-to-stand transitions. Schenkman et al. [10] identified four distinct phases of sit-to-stand movement, with the momentum transfer phase, when body weight shifts from the seated base to the feet, requiring the highest mechanical demands. Riley et al. [11] demonstrated that knee moments during this phase can exceed 1.5 times body weight. Our gas springs are configured to provide maximum assistance during this critical phase, compensating for absent muscle power in individuals with paralysis. Additionally, the forward-leaning posture naturally engages remaining trunk musculature and maintains the user’s center of gravity within a stable base throughout the transition.

The feasibility of this approach is further supported by the low perceived exertion reported by participants. Modified Borg Scale ratings remained low (median 3, range 0–4) across all participants, indicating that the spring assistance effectively reduced physical demands. Previous studies have shown that ratings of perceived exertion in individuals with SCI can reliably reflect exercise intensity [22], suggesting that our participants’ low ratings reflect the actual reduced physical demands rather than altered perception due to SCI. This is particularly important for individuals with limited cardiovascular reserve, as it allows for repeated practice without excessive fatigue.

The wide range of repetitions (5–70) accommodated by the device demonstrates its adaptability to individual capacity. This variability reflects the heterogeneous nature of the SCI population and highlights the importance of self-paced interventions. Our findings directly address barriers identified in our previous survey [8], where 19.4% of individuals discontinued standing training due to difficulties with orthotic devices. The elimination of complex bracing requirements and the intuitive control mechanism reduce both the physical and cognitive demands of standing practice.

The observed changes in MAS scores warrant discussion as an exploratory finding. While the reduction from median 8.75 to 4.25 did not reach statistical significance (*p* = 0.13), the clinical observations were noteworthy. Four of six participants showed reductions in muscle tone, with the most substantial change observed in the participant with the highest baseline spasticity (12.5 to 2). We acknowledge the known limitations of the MAS in SCI populations, with inter-rater reliability reported to be only fair [24]. Despite these limitations, we chose the MAS as it remains the most widely used clinical measure of spasticity. The pattern of immediate tone reduction following voluntary repetitive movements in those with moderate to severe baseline spasticity is consistent with our previous findings [25], suggesting that this approach may offer clinical benefit for selected individuals, though larger studies are needed to confirm these observations.

Despite MASCIP guidelines recommending regular standing [3], the actual implementation remains challenging. While standing frames and tilt tables enable static standing positions, there is a notable absence of devices that facilitate repetitive sit-to-stand training. This gap is particularly significant for individuals with complete paraplegia. Robotic rehabilitation devices have demonstrated functional improvements in individuals with SCI [14,15,27], suggesting that this population retains capacity for recovery even in chronic phases. However, the high cost and complexity of these technologies limit their accessibility in routine clinical practice. The current device addresses these limitations by offering a simpler alternative for repetitive standing practice.

The iterative design process demonstrated in this study highlights the importance of user-centered development in rehabilitation technology. The progression from R1A through R1C addressed specific challenges identified during clinical testing, including transfer difficulties, sitting stability, grip comfort, and assistance adjustment capabilities for physicians and therapists. Intermediate testing with the R1B prototype confirmed improvements in handle comfort and knee stability while identifying the need for further refinements in base height and backrest angle. This approach contrasts with traditional top-down device development and emphasizes the value of early clinical feedback in creating practical rehabilitation solutions. The incorporation of an adjustable assistance mechanism in R1C particularly addresses the heterogeneous nature of the SCI population, where individualized support levels are essential for safe and effective training.

Our findings contribute to addressing the urgent need for accessible standing solutions in aging societies. With over 6200 new SCI cases annually in Japan [2] and limited rehabilitation resources, there is a critical need for accessible standing devices. The Japanese healthcare context presents unique challenges that make simplified standing solutions particularly relevant. After the 180-day acute rehabilitation period covered by national insurance [16], individuals must navigate a complex landscape of long-term care services that prioritize daily living support over therapeutic interventions. Private rehabilitation services, when available, are often financially inaccessible for most individuals without insurance coverage. Furthermore, while the number of rehabilitation professionals in Japan has increased, specialized SCI rehabilitation centers remain unevenly distributed across the country, with many regions lacking accessible facilities. These systemic constraints mean that any viable standing solution must be implementable without constant professional supervision, affordable enough for individual purchase or rental, and simple enough for family caregivers to assist with setup.

The spring-assisted approach offers a middle ground between static standing frames that lack dynamic movement and complex powered systems that remain inaccessible to most individuals. By reducing costs compared to powered alternatives while maintaining safety and effectiveness, this approach may improve access to regular standing practice.

The importance of accessible rehabilitation technologies has gained renewed attention with advances in regenerative medicine. Emerging evidence suggests that the effectiveness of regenerative therapies depends on concurrent rehabilitation to promote neural plasticity and functional recovery [28,29]. The spring-assisted approach demonstrated in this study could provide a practical platform for intensive standing training as these therapies become more widely available.

Several limitations must be acknowledged. The small sample size and absence of a control group limit definitive conclusions about effectiveness. We did not include comprehensive cardiovascular monitoring or detailed biomechanical analysis, which would strengthen understanding of the mechanisms underlying the observed safety and feasibility. The single-session design cannot address questions about training frequency, optimal duration, or long-term adherence. Additionally, recruitment from a single outpatient clinic may have selected for more motivated individuals, potentially overestimating feasibility in the broader SCI population. While the R1A testing included both male and female participants, the R1B evaluation included only male participants due to recruitment constraints during the COVID-19 pandemic. Although the device design is not gender-specific and female participants successfully used the R1A prototype, future studies should ensure balanced gender representation to confirm the generalizability of findings across all potential users.

Furthermore, the absence of electromyographic data limits our understanding of muscle activation patterns during assisted transitions. While we observed low perceived exertion and successful performance across participants, objective measurement of muscle activity would provide valuable insights into the compensatory strategies employed and the actual biomechanical demands placed on remaining musculature. Future studies incorporating EMG analysis could elucidate how individuals with different injury levels utilize their preserved muscle function during spring-assisted standing, potentially informing more targeted rehabilitation strategies.

Despite these limitations, this study provides initial evidence that spring-assisted standing training can address key barriers to standing practice identified in our population. The approach aligns with the realities of the Japanese healthcare system while maintaining the safety necessary for individuals with high-level complete injuries. Although this study focused on individuals with chronic SCI in a single-session evaluation, application to patients in acute and recovery phases could potentially improve trunk function. Furthermore, this approach may benefit individuals with other conditions affecting trunk and lower limb function beyond SCI.

Future studies should explore optimal training parameters, long-term effects, and applications across different phases of rehabilitation and various patient populations. Incorporating EMG analysis could elucidate how individuals with different injury levels utilize their preserved muscle function during spring-assisted standing, which may inform more targeted rehabilitation strategies. This approach could also complement emerging therapeutic interventions, particularly as regenerative medicine advances. As rehabilitation paradigms evolve to emphasize long-term management of chronic conditions, simplified yet effective interventions become increasingly valuable for bridging the gap between clinical recommendations and real-world practice.

The convergence of our clinical safety data with previously reported user perspectives [8] provides a comprehensive foundation for advancing standing rehabilitation in resource-limited settings. Our demonstration of safe standing transitions even in individuals with high thoracic complete injuries addresses critical safety concerns that have historically limited implementation. Furthermore, the trend toward reduced muscle tone observed in participants with baseline spasticity suggests potential for addressing one of the most challenging aspects of chronic SCI management. As healthcare systems globally face increasing demands with aging populations and rising chronic disease burden, the development of user-driven, mechanically simple solutions becomes essential. The spring-assisted approach represents a pragmatic middle ground between ideal therapeutic goals and practical constraints, offering a pathway toward sustainable standing practice that can be maintained throughout the chronic phase of SCI.

## 5. Conclusions

This preliminary study demonstrated that spring-assisted standing training can be safely performed by individuals with chronic SCI, including those with complete thoracic injuries. The device enabled independent sit-to-stand transitions with low perceived exertion. Importantly, iterative prototype testing with patient feedback led to progressive design improvements addressing transfer ease, sitting stability, and therapist assistance capabilities. These results suggest that user-centered development of simplified standing devices may help address barriers to regular standing practice. Larger controlled studies are needed to confirm these observations and establish optimal training protocols.

## 6. Patents

International Publication No. WO 2023/127630, Assistance Device and Moving Device Using Same.

International Publication No. WO 2024/048616, Assistance Device Equipped with Handle and Movement Device Using Same.

## Figures and Tables

**Table 1:** Participant Demographics and Immediate Effects of Qolo Training (R1A).

ID	Sex	Age (Years)	Ht (cm)	Wt (kg)	NLI	AIS	MMT		Reps	Borg Scale		Total MAS *	
							Hip Ext	Knee Ext		Pre	Post	Pre	Post
1	F	23	165	55	T4	A	0	0	5	1	1	12.5	2
2	M	47	165	65	T6	A	0	0	41	0	3	8	8
3	M	33	170	55	T11	A	0	1	33	4	2	0	0
4	M	56	175	65	T9	A	0	0	60	2	3	9	4.5
5	F	35	155	60	T10	A	0	1	20	0	4	10	6
6	M	56	166	59	L3	C	1	3	52	0	3	8	4
Median		41	165.5	60	-	-	0	0.5	37	0.5	3	8.75	4.25
Min-Max		23–56	155–175	55–65	-	-	0–1	0–3	5–60	0–4	1–4	0–12.5	0–8

Abbreviations: Ht = Height; Wt = Weight; NLI = Neurological Level of Injury; AIS = ASIA Impairment Scale; MMT = Manual Muscle Testing (0–5 scale); Reps = Number of sit-to-stand repetitions; MAS = modified Ashworth Scale. * Total MAS represents the sum of all joint measurements (bilateral) with 1+ scored as 1.5.

**Table 2:** Participant Demographics and Immediate Effects of Qolo Training (R1B).

ID	Sex	Age	Ht	Wt	NLI	AIS	MMT		Reps	Borg Scale	
		(Years)	(cm)	(kg)			Hip	Knee		Pre	Post
							Ext	Ext			
2	M	47	165	65	T6	A	0	0	40	0.5	2
3	M	33	170	55	T11	A	0	1	28	-	-
4	M	56	175	65	T9	A	0	0	20	3	4
6	M	56	166	59	L3	C	1	3	70	1	3

Abbreviations: Ht = Height; Wt = Weight; NLI = Neurological Level of Injury; AIS = ASIA Impairment Scale; MMT = Manual Muscle Testing (0–5 scale); Reps = Number of sit-to-stand repetitions. Note: Borg Scale data was not recorded for ID 3.

## Data Availability

The original contributions presented in this study are included in the article. Further inquiries can be directed to the corresponding author.

## Supplementary Materials

The following supporting information can be downloaded at: https://www.mdpi.com/article/10.3390/jcm14196767/s1, Supplementary Table S1: Individual Modified Ashworth Scale (MAS) Scores by Joint and Movement Before and After Spring-Assisted Standing Training.
