# Supplementary material for: Development and Clinical Evaluation of Spring-Assisted Standing Training for Individuals with Spinal Cord Injury: A Safety and Feasibility Study
Source: J Clin Med. 2025 Sep 25;14(19):6767. doi: 10.3390/jcm14196767 (PMC12525290; doi:10.3390/jcm14196767)
Supplement: Supplementary file 1 [file jcm-14-06767-s001.zip › jcm-3839712-supplementary.pdf]

Supplementary Table S1. Individual Modified Ashworth Scale (MAS) Scores by Joint and Movement Before and After Spring-Assisted Standing Training

ID	Sex	Ht (cm)	Wt (kg)	NLI	AIS	MMT		Reps	Borg Scale		MAS Hip Flex				MAS Hip Abd				MAS Knee Flex				MAS Knee Ext				MAS Ankle PF				MAS Ankle DF			
											Pre		Post		Pre		Post		Pre		Post		Pre		Post		Pre		Post		Pre		Post	
						Hip Ext	Knee Ext		Pre	Post	R	L	R	L	R	L	R	L	R	L	R	L	R	L	R	L	R	L	R	L	R	L	R	L
1	F	165	55	T4	A	0	0	5	1	1	1	1	0	0	1	1	0	0	1	1+	0	0	1	1	0	0	0	0	0	0	2	2	1	1
2	M	165	65	T6	A	0	0	41	0	3	1	1	1	1	1	1	1	1	1+	1+	1	1	0	1	1	1	0	0	0	0	0	0	0	0
3	M	170	55	T11	A	0	1	33	4	2	0	0	0	0	0	0	0	0	0	0	0	0	0	0	0	0	0	0	0	0	0	0	0	0
4	M	175	65	T9	A	0	0	60	2	3	1	1	1	1	1	0	0	0	0	0	0	0	1	1	0	0	0	0	0	0	2	2	1+	1
5	F	155	60	T10	A	0	1	20	0	4	1+	1	1	1	1+	1	1	1	1	0	0	0	0	0	0	0	0	0	0	0	2	2	1	1
6	M	166	59	L3	C	1	3	52	0	3	0	1	0	0	0	1	0	0	1	1	0	0	0	1	0	1	0	0	0	0	1+	1+	1+	1+

Abbreviations: ID = Participant identification number; Ht = Height; Wt = Weight; NLI = Neurological Level of Injury; AIS = ASIA Impairment Scale; MMT = Manual Muscle Testing (0-5 scale); Reps = Number of sit-to-stand repetitions; R = Right; L = Left; Hip Flex = Hip flexion; Hip Abd = Hip

abduction; Knee Flex = Knee flexion; Knee Ext = Knee extension; Ankle PF = Ankle plantarflexion; Ankle DF = Ankle dorsiflexion

The table demonstrates the variability in spasticity patterns among participants and the immediate changes observed following the intervention. Four of six participants (ID1, 4, 5, 6) showed reductions in MAS scores across multiple joints, with changes most observed in hip flexion and knee extension movements. One participant (ID3) had no baseline spasticity and remained at zero, while participant ID2 presented with myoclonus-like involuntary movements in addition to spasticity, which may have affected the accuracy of MAS assessment and contributed to the unchanged scores observed in this individual.
